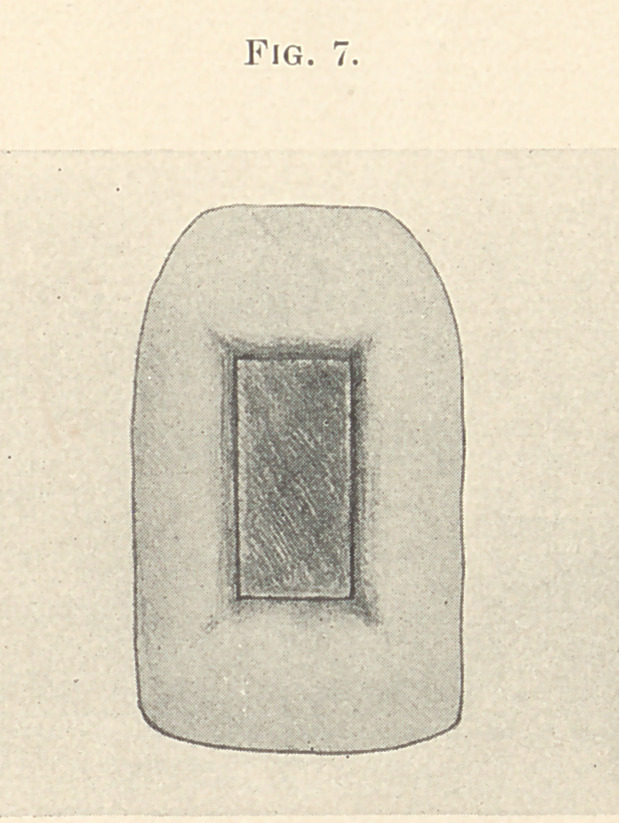# Baked Porcelain Restorations of Broken Bridge Facings

**Published:** 1903-06

**Authors:** Joseph E. Duffield

**Affiliations:** Camden, N. J.


### International Dental Journal.

VOL. XXIV. JUNE, 1903. No. 6.

### Original Communications.[1]

### BAKED PORCELAIN RESTORATIONS OF BROKEN BRIDGE FACINGS.

BY DR. JOSEPH E. DUFFIELD, CAMDEN, N. J.

MANY vain efforts have been made to repair fixed bridges and broken crowns where the facings have split away from the backing, due either to accident or to occlusion which has been too close to permit of proper protection by the usual means. Those attempts have included the Bryant method of cutting a thread on the pins and attaching by means of a nut; cutting a dovetailed cavity in the backing and bending the pins of the tooth so as to key the work in place, and various other systems. Undoubtedly it requires no ordinary skill to so grind a plate tooth that it may approximately fit the backing already in position, and which is invariably of such thickness as to not permit of any adaptation by burnishing.

Again, after the tooth has been ground and fitted to the entire satisfaction of the operator, and the pins of the tooth in question passed through the openings in the backing, which have been very carefully tapped out to accurately receive the same, we so often

---

[1] The editor and publishers are not responsible for the views of authors of papers published in this department, nor for any claim to novelty, or otherwise, that may be made by them. No papers will be received for this department that have appeared in any other journal published in the country.

find the thickness of the backing such as to prevent of sufficient countersinking on the palatal surface, thereby preventing the work from being securely and permanently attached by riveting or by tinning the pins and filling in with amalgam.

To the writer one of the most objectionable features of the plans employed in repairing those conditions has been the utter lack of close adaptation of the parts repaired.

For the following operation which was evolved, and which has proved very satisfactory after a test of now nearly three years, two points of superiority are claimed,—first, perfect adaptation; second, strength; two very essential features, and upon which rests the success of the work.

In the employment of the method about to be described, it is necessary that the pins remain intact in the backing. After clearing away all particles of porcelain, which may be adhering to the pins from the fractured facing, a cement filling is built around the same, making the sides parallel, the cement extending in a lateral direction only far enough to include the overhang of the pin-heads, the filling being flush with the tops of the pins; the object being to permit of the free drawing of the matrix about to be made. (Fig. 1.)

Platinum-foil, gauge $^{1}/_{1000}$, is then burnished over the entire backing, the cement filling being permitted to protrude through the platinum-foil and extend well up on the adjacent teeth. (Fig. 2.)

The matrix is then removed and laid aside, and a small piece of foil, cut oblong and sufficiently large to cover the cement filling and extend down the sides of the same to the backing, is then prepared by slitting from the four corners. (Fig. 3.) The object is to burnish the foil over the filling and form a box without tearing (Fig. 4.)

With the box still in position, the matrix already described is again placed on the backing and the two pieces joined with a small amount of paraffin. The entire work is lifted off and the paraffin eliminated by holding in a flame; a quantity of tooth body of the desired shade is then placed on the matrix and fused. (Fig. 5.)

There now being no danger of destroying the perfect adaptation by handling, it is again placed in position on the backing and a porcelain veneer or plate tooth, from which the pins have been removed, and of a proper shade, is ground in position. Additional tooth body is added to the matrix and the under side of the veneer,

Fig. 1.

Fig. 3.

Fig. 2.

Fig. 4.

Fig. 5.

Fig. 7.

Fig. 6.

which is then placed on the matrix and gently pressed in position. A few blasts of hot air are applied to carry off the superfluous moisture in the body, and with an excavator the matrix and veneer, as one piece, are gently lifted off the backing and allowed to fall on a doily. (Fig. 6.) It is then placed in the furnace and fused. The platinum-foil is stripped off from the back, and with a small diamond disk the box or countersunk cavity in the porcelain is undercut. The cement about the pins in the backing is removed, and the work is ready for final cementation to position. (Fig. 7.)

If the operation has been carefully executed, we now have a repair which in point of contour and adaptation is eminently satisfactory, and one which is approximately as strong as the original.

## Figures and Tables

**Fig. 1. f1:**
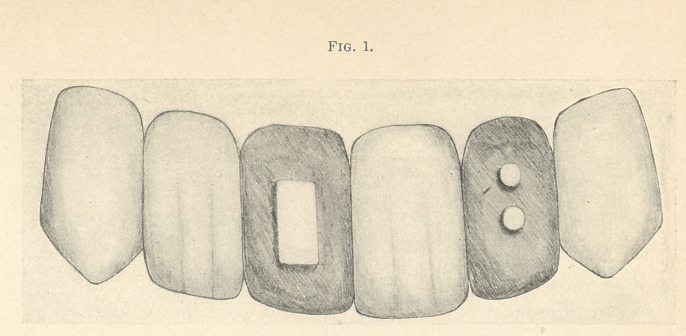


**Fig. 2. f2:**
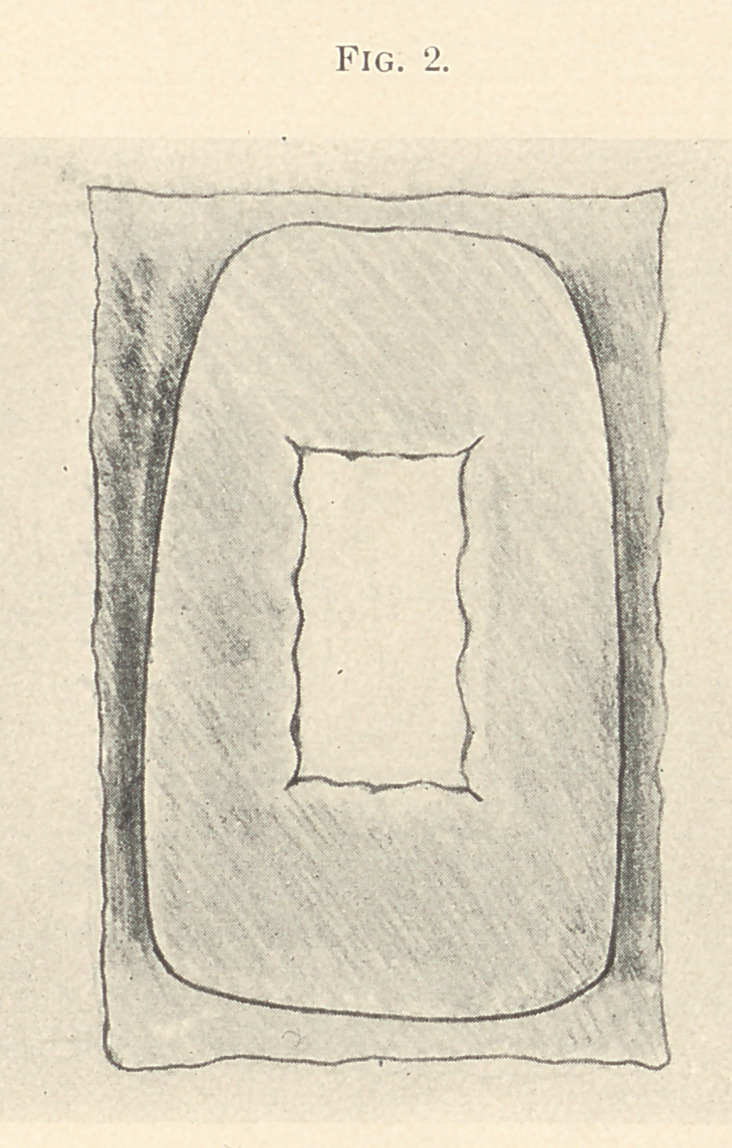


**Fig. 3. f3:**
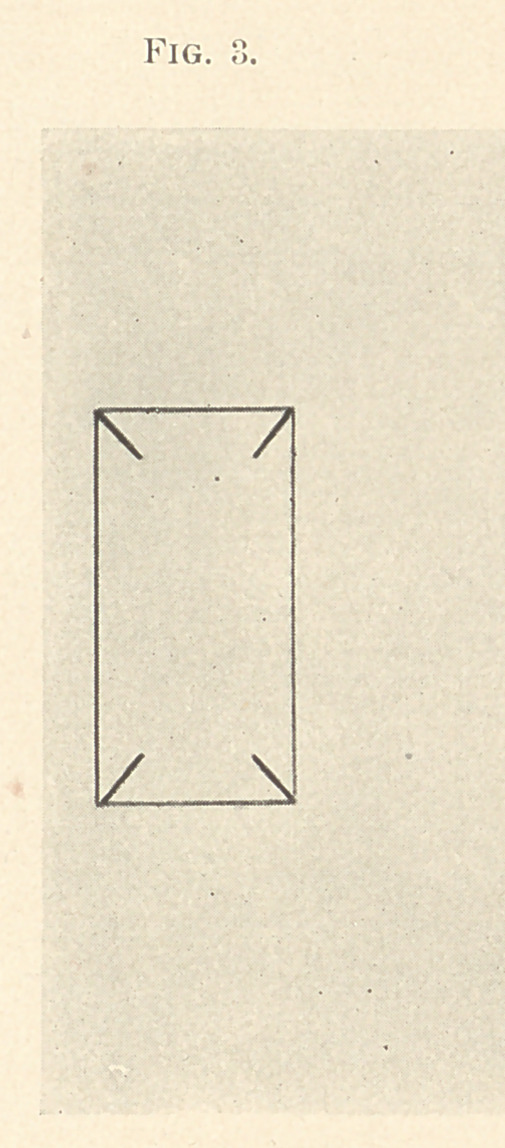


**Fig. 4. f4:**
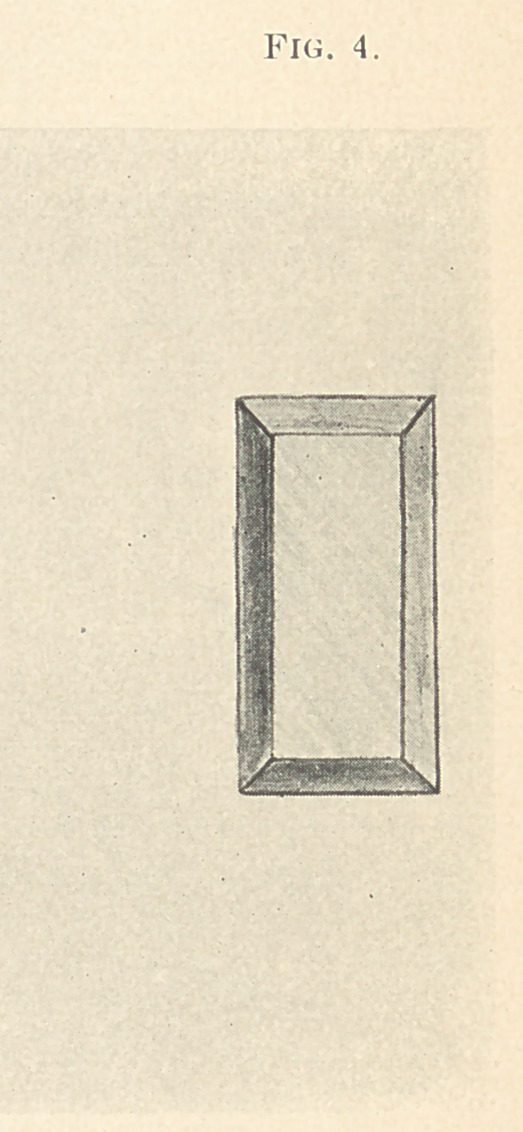


**Fig. 5. f5:**
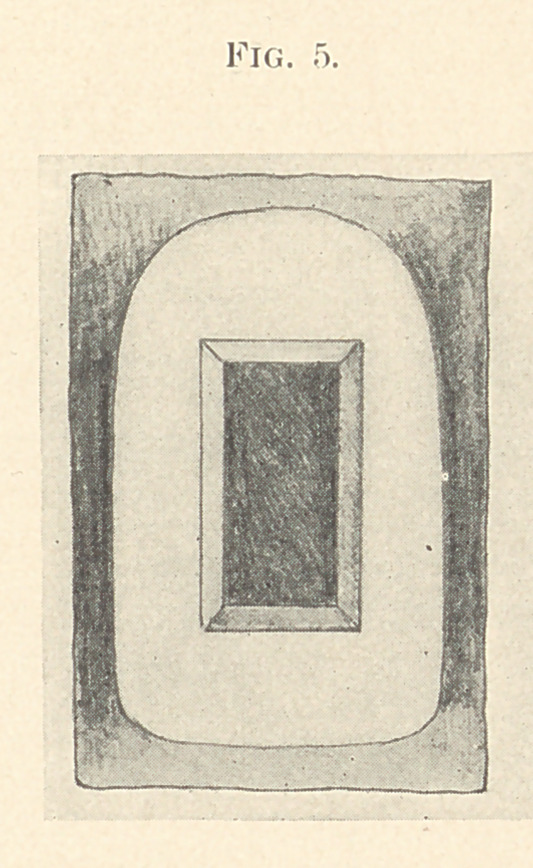


**Fig. 6. f6:**
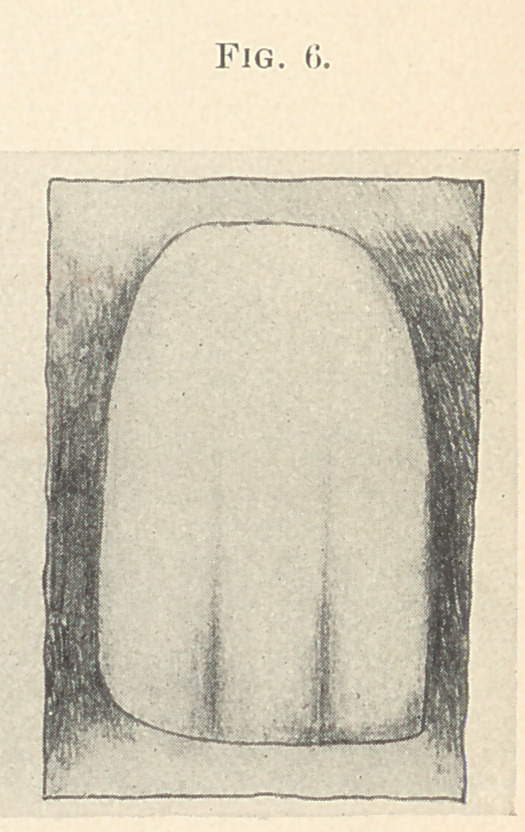


**Fig. 7. f7:**